# Antioxidant Properties of Olive Mill Wastewater Polyphenolic Extracts on Human Endothelial and Vascular Smooth Muscle Cells

**DOI:** 10.3390/foods10040800

**Published:** 2021-04-08

**Authors:** Anna Maria Posadino, Annalisa Cossu, Roberta Giordo, Amalia Piscopo, Wael M. Abdel-Rahman, Antonio Piga, Gianfranco Pintus

**Affiliations:** 1 Department of Biomedical Sciences, University of Sassari, 07100 Sassari, Italy; posadino@uniss.it (A.M.P.); cossuannalisa@libero.it (A.C.); 2 Department of Medical Laboratory Sciences, Institute for Medical Research, College of Health Sciences and Sharjah, University of Sharjah, Sharjah P.O. Box 27272, United Arab Emirates; robertagiordo2000@yahoo.it (R.G.); whassan@sharjah.ac.ae (W.M.A.-R.); 3 Department of AGRARIA, Mediterranean University of Reggio Calabria, 89124 Vito Reggio Calabria, Italy; amalia.piscopo@unirc.it; 4 Department of Agricultural Environmental Sciences and Food Biotechnology, University of Sassari, Viale Italia 39, 07100 Sassari, Italy

**Keywords:** antioxidant activity, olive mill wastewaters, phenolic extract, reactive oxygen species, vascular cells

## Abstract

This work aims to analyze the chemical and biological evaluation of two extracts obtained by olive mill wastewater (OMW), an olive oil processing byproduct. The exploitation of OMW is becoming an important aspect of development of the sustainable olive oil industry. Here we chemically and biologically evaluated one liquid (L) and one solid (S) extract obtained by liquid–liquid extraction followed by acidic hydrolysis (LLAC). Chemical characterization of the two extracts indicated that S has higher phenol content than L. Hydroxytyrosol and tyrosol were the more abundant phenols in both OMW extracts, with hydroxytyrosol significantly higher in S as compared to L. Both extracts failed to induce cell death when challenged with endothelial cells and vascular smooth muscle cells in cell viability experiments. On the contrary, the higher extract dosages employed significantly affected cell metabolic activity, as indicated by the MTT tests. Their ability to counteract H_2_O_2_-induced oxidative stress and cell death was assessed to investigate potential antioxidant activities of the extracts. Fluorescence measurements obtained with the reactive oxygen species (ROS) probe H_2_DCF-DA indicated strong antioxidant activity of the two OMW extracts in both cell models, as indicated by the inhibition of H_2_O_2_-induced ROS generation and the counteraction of the oxidative-induced cell death. Our results indicate LLAC-obtained OMW extracts as a safe and useful source of valuable compounds harboring antioxidant activity.

## 1. Introduction

The olive oil industry is a large productive sector globally, and three-quarters of world production takes place in Europe. Among different commercial categories, extra virgin olive oil (EVOO) is produced by the sole employment of mechanical processes, and its quality is tightly related to different parameters, such as agronomic practices, the olive cultivar, and the olive oil extraction technology used [1]. The final chemical composition of the EVOO is influenced by different factors, among which the olive variety is the first one to play a primary role across the supply chain. Indeed, the olive cultivars display a large range of genetic variability for several agronomic traits such as the fruit size, oil content, and the degree of adaptation to severe environmental stress [2,3]. Olive drupes possess various health-promoting bioactive substances primarily represented by biophenol secoiridoids (oleuropein, ligstroside) and their hydrolytic derivatives [4]. Although olive fruit is rich in phenolic compounds, only 2% of the entire phenolic content transfers into the oil phase, while the rest goes into the olive mill wastewater (OMW) (approximately 53%), the pomace, the olive oil filtration residue, and olive leaves (approximately 45%) [5]. As a result, the high percentage of phenolic compounds in the olive industry’s byproducts is attracting great interest as a potential source of such phenolic compounds, with special attention oriented to the olive mill, which is the primary potential source of such molecules. The major components in OMW include hydroxytyrosol, tyrosol, oleuropein, ligstroside and their secoiridoids derivatives, and a variety of hydroxycinnamic and hydroxybenzoic acids [6]. Given the potential environmental impact, active molecule extraction from olive oil byproducts should embrace methodologies that employ green technologies, considering their possible exploitation as food antioxidants or nutraceuticals [7]. In fact, phenol recovery from these byproducts should be organized in order to promote their reintroduction into the food chain and coincide with greater valorization and improved olive oil industry waste management. In addition to its main implications for the local and international economy, this could reduce the environmental impact of olive oil manufacturing and contribute to this valuable production chain’s sustainability. However, the quality of OMW phenolic compounds differs according to several factors, including the olive oil production technological process employed, and for this reason, it is essential to evaluate different OMW technological processes to provide promising bioactive compounds. In this regard, OMW-derived products have been tested for certain biological effects and have showed an interesting bioactivity spectrum [8]. For instance, EVOO-containing phenolic compounds have shown In Vivo and In Vitro antioxidant activity, likely due to molecules such as hydroxytyrosol, tyrosol, and secoiridoid derivatives [9,10].

Reactive oxygen species (ROS) are aerobic metabolism products and exert a pivotal role in regulating cellular functions such as proliferation, differentiation, and migration [11]. Under physiological conditions, a series of cellular antioxidant mechanisms maintain vascular ROS levels in homeostatic conditions [12]. However, an aberrant modulation of the above-mentioned mechanisms leads to critical increases in ROS levels, thus promoting different vascular-associated pathological conditions, including cancer and cardiovascular diseases [13]. In this context, endogenous ROS, released by endothelial and vascular smooth muscle cells as a result of pro-inflammatory and pro-atherosclerotic stimuli, can trigger vascular injury and blood vessel restructuring by affecting diverse intracellular signaling pathways [14]. Indeed, ROS-activated molecular machinery can modulate both endothelial cells and vascular smooth muscle cells functions including proliferation, migration, and invasion, leading to vascular pathologies such as hypertension, atherosclerosis, and cancer [15], which may be counteracted by OMW-contained compounds [16].

In this light, the present work aims to investigate whether (i) the considered OMW samples can be a source of valuable antioxidants, and (ii) whether the obtained extracts can protect human vascular cells against oxidative cell death.

## 2. Materials and Methods

### 2.1. Chemicals

Unless stated in the text, all the reagents used were from Sigma (Sigma, St. Louis, MO, USA).

### 2.2. Sample Collection

Ottobratica olives were sampled in November 2019 at a ripening index of 4 according to Guzmán et al. [17]. Oil extraction was performed by means of a three-phase decanter system (Alfa Laval, Monza, Italy) at 25–26 °C and 20 min of malaxation parameters in an olive oil mill located in the Calabria region (Italy). The obtained OMWs were transferred to the Food Technologies laboratory of the Mediterranea University of Reggio Calabria for the experimental project.

### 2.3. OMW Acquisition and Preparation

The extract was obtained following the method reported by De Marco et al., with some modifications [18]. Two liters of olive oil mill wastewater (OMW) were acidified to pH 2 with HCl and washed three times with hexane (1:1, *v*:*v*) in order to remove the lipid fraction. The mixture was vigorously shaken and centrifuged under 3000 rpm for 3 min at 10 °C. The phenolic compounds were extracted by mean of ethyl acetate three times in a separating funnel (1:4 *v*:*v*), and then the combined extracts were centrifuged for 5 min at 3000 rpm at 10 °C. The organic phase was separated and filtered through a sintered glass Buchner apparatus. Then the ethyl acetate was evaporated under vacuum using a rotary vacuum evaporator at 25 °C (headspace analysis was performed). Finally, the dry residue was again dissolved in 100 mL of water, filtered using PTFE 0.45 μm (diameter 15 mm) syringe filter, and stored at 4 °C until subsequent analyses. An aliquot of the obtained extract (called L) was freeze-dried in a VirTis lyophilizer (Gardiner, NY, USA) and the obtained sample was called S. For all the experiments, we used both types of phenolic extracts, the freeze-dried (S) and the wet (L) type.

### 2.4. Determination of Total and Individual Phenolic Content of OMW Polyphenolic Extracts

The total phenol content was determined spectrophotometrically as previously described by Bruno et al. with some modifications [19]. An aliquot portion (0.1 mL) of phenolic extract was placed in a 25 mL volumetric flask and mixed with 20 mL of deionized water and 0.625 mL of the Folin–Ciocalteau reagent. After 3 min, 2.5 mL of saturated solution of Na_2_CO_3_ (20%) was added. After that, the mixture was incubated for 12 h at room temperature and in the dark. The sample’s absorbance was measured at 725 nm against a blank and compared with a gallic acid (GA) calibration curve (concentration between 1 and 10 mg L^−1^). The results were expressed as mg of GA g^−1^ of phenolic extract.

Identification and quantification of S and L extracts’ main phenolic compounds were performed by HPLC-DAD (Dionex Ultimate 3000 RSLC, Waltham, MA, USA), as previously described [20]. The phenolic determination was conducted using the Dionex Acclaim 120 C18 analytical column (3 µm, 150 × 3 mm) (Thermo Scientific, Waltham, MA, USA) set at 35 °C, a flow rate of 1 mL min^−1^, and an injection volume of 5 µL. Water/acetic acid (98:2, *v*/*v*) (A) and acetonitrile (B) were used as mobile phases, and the applied gradient was the following: 95% A and 5% B (5 min), 80% A and 20% B (10 min), 75% A and 25% B (15 min), 65% A and 35% B (20 min), 0% A and 100% B (25 min), and 95% A and 5% B (35 min). Quantification was performed by pure standard (Sigma-Aldrich Co. LLC, St. Louis, MO, USA) and data were expressed as mg g^−1^ of phenolic extract.

### 2.5. Cell Culture

Human umbilical vein endothelial cells (HUVECs) were acquired from Cell Application (San Diego, CA, USA) and grown as previously described [21]. Briefly, cells were grown in endothelial cell basal medium supplemented with cell growth supplement (EGM-V2 # 213K-500) as per company instructions. When confluent, cells were sub-culture at a split ratio of 1:2 and used within three passages. Unless specified in the text, cells were plated in 96-well plates (Corning, Lowell, MA, USA) at a concentration of 10^5^ cells/mL and processed for experiments in a complete medium containing the different concentrations of the extracts.

Human pulmonary artery smooth muscle cells (HPASMCs) were acquired from Cell Application (San Diego, CA, USA) and grown as previously described [22]. Briefly, cells were grown in smooth muscle cells basal medium supplemented with cell growth supplement (HSMCs-kit #311K-500) as per company instructions. When confluent, HSMCs were sub-culture at a split ratio of 1:2 and used within three passages. Unless specified in the text, cells were plated in 96-well plates (Corning, Lowell, MA, USA) at a concentration of 10^5^ cells/mL and processed for experiments in a complete medium containing the different concentrations of the extracts.

According to data obtained in our previous studies [23,24,25,26], we decided to test the extracts at the doses of 10, 25, 50, and 100 µg/mL.

### 2.6. Cell Metabolic Assay

Cell metabolic activity was evaluated using the oxidizable and reducible colorimetric probes 3-(4,5-dimethylthiazol-2-yl)-2,5-diphenyltetrazolium bromide (MTT) [23]. Cells were treated as indicated in figure legends and then processed for the MTT assay. After treatments, cells were added with 20 µL MTT solution (5 mg/mL) in medium M199 and placed at 37 °C in a cell incubator for 4 h. After that, the medium was discarded, and the converted dye was solubilized with acidic isopropanol (0.04 N HCl in absolute isopropanol), and the multi wells were read at 570 nm using a GENios plus microplate reader (Tecan) with background subtraction at 650 nm. Results were expressed as a percent of untreated control cells. Cell viability was calculated by the following equation: Cell viability (%) = (Abs of sample/Abs of control) × 100, where Abs of sample is the absorbance of the cells incubated with the different concentrations of the two extracts, and Abs of control is the absorbance of the cells incubated with the culture medium only (positive control).

### 2.7. Measurement of Intracellular ROS

Intracellular ROS levels were determined by using the ROS molecular probe 2′,7′-dichlorodihydrofluorescein diacetate (H_2_DCF-DA) (Molecular Probe, Eugene, OR, USA) as previously described with minor modification [24,25]. In this assay, ROS oxidize H_2_DCF, producing the fluorescent compound DCF, the fluorescence levels of which are proportional to the amount of intracellular ROS. Cells were treated as indicated in figure legends and then processed for the intracellular ROS assessment. For the ROS assay, cells were incubated for 30 min with Hank’s Balanced Salt Solution (HBSS) containing 5 µM H_2_DCF-DA, then washed twice with HBSS, and then fluorescence was measured by using a GENios plus microplate reader (Tecan, Mannedorf, Switzerland. Excitation and emission wavelengths used for fluorescence quantification were 485 and 535 nm, respectively. All fluorescence measurements were corrected for background fluorescence and protein concentration. Using untreated cells as a reference, the antioxidant and prooxidant outcomes were evaluated by comparing five measurements and expressed as a percentage of untreated control cells.

### 2.8. Cell Viability Assay

Cell viability was assessed as previously described [26] by using the CytoTox-ONE™ (Promega, Madison, WI, USA) kit. The CytoTox-ONE™ homogeneous membrane integrity assay is a fluorometric method for estimating the number of nonviable cells present in multi-well plates. The assay measures the release of lactate dehydrogenase (LDH) from cells with a damaged membrane. Then the LDH released into the culture medium is measured with a 10-min coupled enzymatic assay that results in the conversion of resazurin into a fluorescent resorufin product. The amount of fluorescence produced is proportional to the number of dead cells with the lysed membrane.

Cells were treated as indicated in figure legends and then processed as per company instructions at each experimental point’s end. Fluorescence was measured by using a GENios plus microplate reader (Tecan, Mannedorf, Switzerland). Excitation and emission wavelengths used for fluorescence quantification were 535 and 620 nm, respectively. All fluorescence measurements were corrected for background fluorescence and protein concentration, and results were expressed as a percentage of untreated control cells.

### 2.9. Statistical Analysis

Data were expressed as means ± S.D. of the indicated number of experiments. One-way analysis of variance (ANOVA) followed by a post-hoc Newman–Keuls multiple comparison test were used to detect differences of means among treatments with significance defined as *p* < 0.05. Statistical analysis was performed using GraphPad Prism version 8.00 for Windows, GraphPad Software, San Diego, CA, USA.

## 3. Results

In this work, two extracts derived from OMW were assessed for their phenol contents and their potential biological activity on two human vascular cells. The two extracts were obtained employing a desolvented ethyl acetate extraction followed by water recovery.

**Figure 1:**
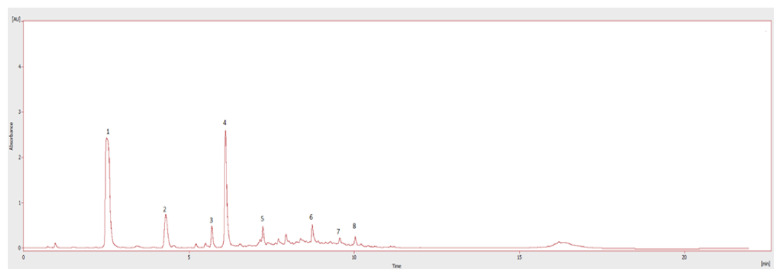
**Chromatogram of phenolic compounds in S olive mill wastewater (OMW) extract**. (1) Hydroxytyrosol; (2) tyrosol; (3) vanillic acid; (4) caffeic acid; (5) ferulic acid; (7) apigenin-7-O-glucoside; (8) oleuropein; (9) luteolin-7-O-glucoside.

Hydroxytyrosol and tyrosol were the principal quantified compounds in both OMW extracts, with significantly greater abundance in the S extract, mainly related to the first phenolic alcohol (4.55 mg g^−1^). The tyrosol concentration varied between 0.54 mg g^−1^ in L extract and 0.58 mg g^−1^ in L extract. The S extract also showed higher amounts of caffeic acid and oleuropein, respectively, of 0.36 and 0.15 mg g^−1^. Finally, among the identified flavonoids, apigenin-7-O-glucoside was more abundant in L extract with a concentration of 0.15 mg g^−1^ (Figure 1).

Next, we proceeded with the extracts study by assessing their potential biological activities on two human vascular cells, HUVECs and HSMC, respectively, endothelial and vascular smooth muscle cells. Based on data from our previous studies [27,28,29,30], cell treatments were performed using the following extracts’ concentrations: 10, 25, 50, and 100 µg/mL.

**Figure 2:**
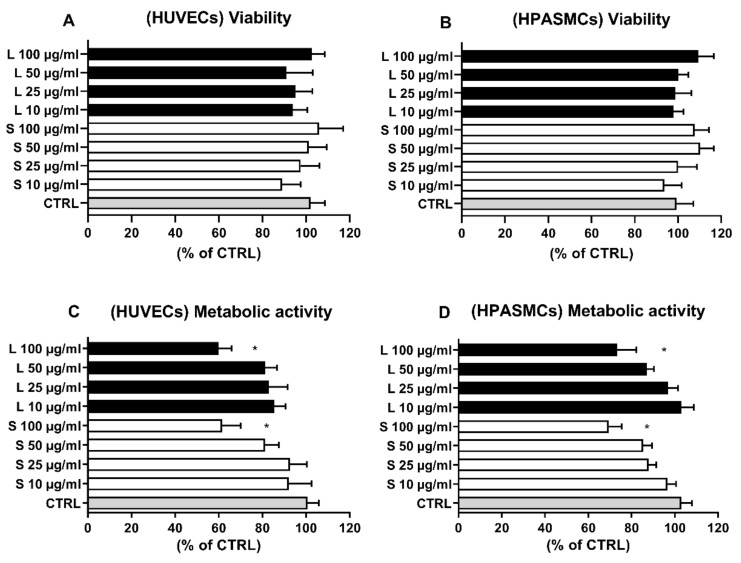
**Effect of OMW on HUVECs and HPASMCs viability and metabolic activity**. Cells were exposed for 24 h in the absence (CTRL) or presence to the indicated concentrations of OMW extracts. Cell viability (**A**,**B**) and metabolic activity (**C**,**D**) were assessed as reported in the materials and methods. HUVECs, human umbilical vein endothelial cell; HPASMCs, human pulmonary artery smooth muscle cells; L, liquid extract; S, solid extract; CTRL, untreated cells; * significantly different from CTRL; Values are shown as mean ± SD and expressed as a percentage of the CTRL. (*n* = 4).

Although widely accepted as a provider of health benefits, natural antioxidants, including phenolic compounds, based on both dosages and redox environment status, have been reported to act as prooxidants, thus increasing intracellular ROS levels and inducing cell death [23,25,26,31,32,33,34]. Moreover, extracts deriving from food processing waste have been reported to be unsafe for the environment [7]. For this reason, we first investigated the potential harmful effects of OMW extracts by assessing their ability to affect cell viability, metabolic activity, and intracellular ROS production, three aspects tightly interconnected in maintaining the physiological functions of vascular cells [35,36]. As reported in Figure 2, 24-h exposition of HUVECs (Figure 2A) and HSMCs (Figure 2B) to the indicated concentrations of OMW failed to induce cell death in either cellular model. To further investigate potential harmful effects and corroborate the absence of cell toxicity, we assessed the extract’s effect on cellular metabolic activity. Figure 2C,D indicate that only the highest dose tested (100 µg/mL) was able to significantly affect the cell metabolic activity, while no effects were observed at the other tested concentrations.

**Figure 3:**
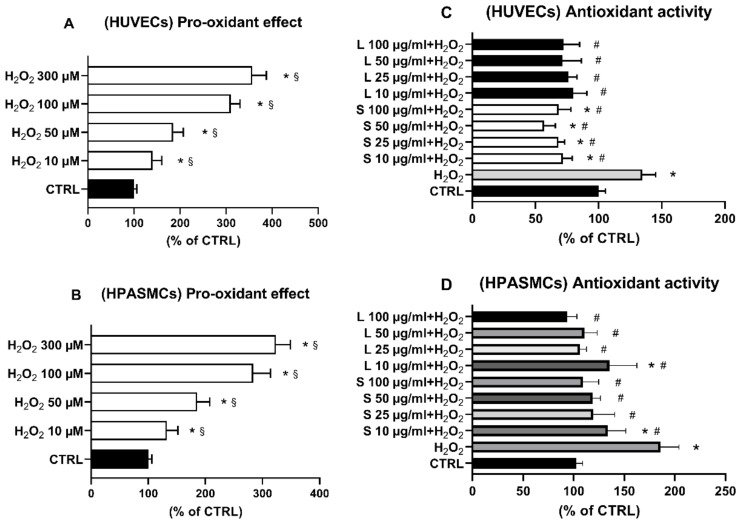
(**A**,**B**) **Dose–response effect of H_2_O_2_ on intracellular ROS levels of HUVECs** (**A**) **and HPASMCs** (**B**)**.** Cells were exposed for 6 h to the indicated concentrations of H_2_O_2_. Intracellular ROS levels were assessed as reported in Materials and Methods. (**C**,**D**) OMW extracts counteract H_2_O_2_-induced ROS increase in both HUVECs (**C**) and HPASMCs (**D**). Cells were exposed for 3 h to the indicated concentrations of OMW extracts and then incubated for 6 h in the presence of 75 µM H_2_O_2_. Intracellular ROS levels were assessed, as reported in Materials and Methods. HUVECs, human umbilical vein endothelial cell; HPASMCs, human pulmonary artery smooth muscle cells; L, liquid extract; S, solid extract; CTRL, untreated cells; H_2_O_2_, hydrogen peroxide; * significantly different from CTRL; § significantly different from each other; # significantly different from H_2_O_2_. Values are shown as mean ± SD and expressed as a percentage of the CTRL. (*n* = 5).

Since the two OMW extracts showed a remarkable quantity of antioxidant phenolic compounds, we sought to investigate their potential antioxidant activity by assessing the ability to counteract H_2_O_2_-elicited oxidative changes. First, we evaluated the ability of our ROS assay to reveal intracellular ROS level variations within a range of selected H_2_O_2_ concentrations. As reported in Figure 3A,B, the probe displayed both a significant dynamic range and linear response when challenged with increasing concentrations of H_2_O_2_, a well-known cellular prooxidant in these vascular cells. [37,38]. Therefore, based on these results, 75 µM was the dosage of H_2_O_2_ employed for the experiments concerning potential extract antioxidant activity. In this experiment, cells were treated for 4 h with the four extract concentrations followed by a 2 h-treatment with 75 μM H_2_O_2_. Then, the levels of intracellular ROS were measured as indicated in the Section 2.

Exposure of the HUVECs to increasing concentrations of the two extracts significantly counteracted the H_2_O_2_-elicited increase of ROS in all the tested concentrations (Figure 3C). Similarly, compared to H_2_O_2_-treated cells, exposure of HSMCs to the two extracts induced a significant antioxidant effect in the whole concentration range (Figure 3D). Since oxidative stress is recognized to cause cell injury and even death, we next hypothesized that the observed extracts’ antioxidant effects could provide cellular protection against cells damaged elicited by H_2_O. To this end, cells were pre-treated for 3 h with the indicated extract concentrations, and then 75 µM H_2_O_2_ was added for 24 h before cell viability determination. In agreement with the displayed antioxidant effect on H_2_O_2_-increased ROS production (Figure 3C,D), cell pre-treatments with increasing concentrations of the two OMW extracts significantly protected both cell lines from H_2_O_2_-elicited oxidative cell death.

## 4. Discussion

World globalization requires sustainable growth. In this context, the processing of different types of waste, derived from food treatment or processing, to produce value-added products is becoming a challenging reality that we must pursue. Waste processing or transformation may involve using different technologies, and the obtained products may or may not be safe for the environment and human health. However, since transformation may give rise to new, unknown, or unwanted compounds, it is useful to assess their safety, especially in terms of their impact on human health.

The olive (*Olea europaea L*.) is native to the Mediterranean area where it can be found in the wild form in the Middle East. *Olea europaea* L. is widely spread throughout the world, especially in the Mediterranean regions, where it accounts for nearly 96 percent of global olive production (FAOSTAT Food and Agriculture Data [20]). The production of olive oil forms a significant quantity of residue, such as aqueous waste called olive mill wastewater (OMW), and olive leaves. Other wastes are also produced during the filtration processes and during the storage time when the solid components migrate to the tank bottoms, generating sediments. These waste products are rich in bioactive compounds and can be therefore considered valuable byproducts to be exploited for further uses [39,40]. Although OMWs are effluents capable of degrading soil and water quality, with serious negative effects on the environment [7], studies have revealed that they contain high concentrations of antioxidants, such as phenolic molecules [41]. However, since the quality of OMW compounds differs according to the olive variety, the technological process of olive oil production, storage time, and climatic conditions [40], it is imperative to evaluate the technological processes employed in OMW processing in order to obtain valuable bioactive compounds. In this regard, phenolic compounds derived from OMW have been reported to show interesting biological properties [10,39,40]. Nonetheless, yet a large amount of crusher residues remain without actual application, since only small quantities are used as natural fertilizers, biomass fuel, additives in animal feed, and activated carbon. However, these residues are a precious starting material to produce extracts of phenolic molecules that could be used in various industrial fields [7]. Different technologies are proposed rather than classical solid–liquid extraction to enhance phenolic compounds’ extraction from different olive oil byproducts.

We used a liquid–liquid extraction procedure followed by acidic hydrolysis [18], which provided two extracts, L and S, harboring a remarkable amount of total phenol content. The quantitative analysis indicated the S extract had a higher phenol content compared to the L extract. Moreover, hydroxytyrosol and tyrosol had the primary phenols in both OMW extracts, with hydroxytyrosol significantly more abundant in the S extract than the L extract (Table 1).

In the cellular experiments, we first evaluated potential extracts’ harmful effects by treating endothelial and smooth muscle cells with different OMW dosages for 24 h. As reported in Figure 2A,B, the extracts were unable to induce cell death at all the concentrations tested. However, sometimes compound effects may affect cell metabolism without resulting in cell death. For this reason, we also assessed the extract effect on the two cells’ metabolic activities. In this analysis, the extract showed no effect, except for the highest concentration, 100 µg/mL, where we found a slight but significant decrease in cell metabolic activity, an aspect that requires further investigations as it has been reported for other phenol compounds [23,25,26,31,32,33,34]. However, the finding that the concentration of 100 µg/mL affects only the cell metabolism without inducing cell death indicated that the extracts, even at high concentrations, appeared not to be toxic, being unable to induce cell death. Considering hydroxytyroso as the main compound present in the OMW, our findings align with In Vivo experiments reporting no toxic effects in rodents fed with HT concentrations as high as 2 g/kg b.wt. [42,43]. Similarly, no adverse clinical, biochemical, or gross necropsy effects were also reported in rats orally administered with HIDROX^TM^, a hydrolyzed aqueous olive pulp extract with a phenol composition closer to our OMW [44]. Moreover, In Vitro experiments, performed with different cell lines, also confirmed our findings reporting lack of cytotoxicity with HT concentrations as high as 500 µM [45,46,47].

**Figure 4:**
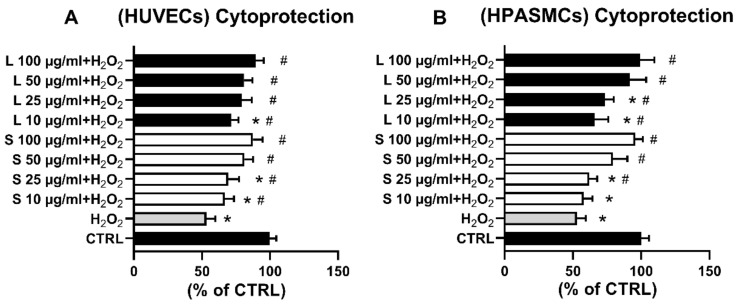
**OMW extracts protect HUVECs** (**A**) **and HPASMCs** (**B**) **from H_2_O_2_-induced cell death**. Cells were exposed for 3 h to the indicated concentrations of OMW extracts and then incubated in the absence (CTRL) or presence of 75 µM H_2_O_2_. Cell viability was assessed as reported in Materials and Methods. HUVECs, human umbilical vein endothelial cell; HPASMCs, human pulmonary artery smooth muscle cells; L, liquid extract; S, solid extract; CTRL, untreated cells; H_2_O_2_, hydrogen peroxide; * significantly different from CTRL; # significantly different from H_2_O_2_. Values are shown as mean ± SD and expressed as a percentage of the CTRL. (*n* = 4).

Although chemical tests such as TBARS, ABTS, and DPPH are often used to determine compounds’ antioxidant properties, a likely better method to investigate the antioxidant activity/effect of a molecule or extract would be In Vivo or in a cellular model. Indeed, a cellular model would provide the proper environment for studying potential interactions (e.g., compound(s)–cellular signal transduction pathway(s), compound(s)–cellular receptor(s)) that would be otherwise missed in a solely chemical setting. In this regard, the intracellular oxidation of H_2_DCF-DA is mainly caused by H_2_O_2_, an oxidizing molecule physiologically present in the cell and therefore functional to induce oxidative insults and study their effects on cell functions [48,49]. Thus we created H_2_O_2_-induced oxidative stress and investigated whether the extracts could counteract it by following the variation of intracellular levels of DFC, the oxidized form of H_2_DCF-DA, and assessing cell viability (Figure 3 and Figure 4). In our experimental conditions, the different extracts’ concentrations significantly inhibited the H_2_O_2_-induced increase of ROS in both HUVECs (Figure 3C) and in HAPVSCs (Figure 3D), bringing the oxidative stress values back to those of the controls. Consonant with observed extracts’ antioxidant activity are the findings reported in Figure 4. The extracts were indeed capable of dose-dependently counteracting H_2_O_2_-induced cell death and restoring cell viability completely at the dosages of 50 and 100 µg/mL. Our current findings support and confirm previously performed experiments; indeed, similarly to EVOO-contained phenolic compounds [9,10,45,46,50], olive oil processing byproducts such as OMW are a source of valuable compounds harboring health benefit properties.

## 5. Conclusions

The OMW extracts obtained by the production of olive oil showed interesting antioxidant activity on both the employed cell models. The tested extracts were capable of effectively protecting cells from oxidative stress-indued cell death, failing indeed to interfere with cell viability and even with the metabolism, except for the highest tested concentration. Our results indicates a different point of view concerning the food processing residues, which should not be considered waste but precious material containing molecules capable of modulating essential functions of cellular biological models, such as the vascular model. Further studies are required to establish these substances’ fates in the organism and to understand their biological functions and fine molecular mechanisms.

## Figures and Tables

**Table 1:** Main phenolic compounds in liquid (L) and freeze dried (S) OMW extracts (mg g^−1^).

Phenolic Compound	L	S	Sign.
Hydroxytyrosol	1.55 ± 0.01	4.55 ± 0.23	**
Tyrosol	0.54 ± 0.01	0.58 ± 0.00	*
Vanillic acid	0.15 ± 0.00	0.09 ± 0.00	**
Caffeic acid	0.22 ± 0.00	0.36 ± 0.00	**
Ferulic acid	0.02 ± 0.00	0.00 ± 0.00	**
Apigenin-7-O-glucoside	0.15 ± 0.00	0.06 ± 0.00	**
Oleuropein	0.08 ± 0.00	0.15 ± 0.00	**
Luteolin-7-O-glucoside	0.04 ± 0.00	0.05 ± 0.00	**

Data are mean (*n* = 2) ± standard deviation. ** Significance for *p* < 0.01; * significance for *p* < 0.05.

## Data Availability

All the data are reported in the article.
